# Surgical Excision of Intramuscular Sarcomas: Description of Three Cases in Dogs

**DOI:** 10.3390/ani13020218

**Published:** 2023-01-06

**Authors:** Matteo Olimpo, Paolo Buracco, Erica Ilaria Ferraris, Lisa Adele Piras, Lorella Maniscalco, Davide Giacobino, Andrea Degiovanni, Emanuela Morello

**Affiliations:** Department of Veterinary Science, University of Turin, 10045 Grugliasco, Italy

**Keywords:** compartmental surgery, local/minimally invasive surgery, intracompartmental muscular sarcomas, dog, surgical oncology

## Abstract

**Simple Summary:**

Local control of intramuscular sarcomas of the limbs may include limb amputation or marginal excision plus radiotherapy. In human medicine, compartmental excision for local control of muscular sarcomas has been widely reported. This procedure is effective when a specific tissue, such as the fascia, acts as a barrier to the neoplastic invasion. The barriers define a compartment, i.e., an anatomo-functional region having the same origin. The aim of this study was to describe this surgical procedure in three dogs affected by intramuscular sarcomas with no signs of fascial invasion at imaging. No major complications were observed in any of the patients, and all experienced a rapid recovery. Tumours treated via compartmental surgery were: chondrosarcoma, a perivascular wall tumour and a hemangiosarcoma. Accurate preoperative tumour staging, including advanced imaging techniques, are necessary in order to perform this procedure successfully.

**Abstract:**

Compartmental excision consists of the complete resection of an anatomic district in which specific structures act as a barrier to local tumour invasion. It is a well-established procedure in human medicine, while only a few reports are available in veterinary medicine. The aim of this study was to describe complete muscle resection in 3 dogs affected by different intramuscular sarcomas. The clinical outcome was also reported. Medical records were searched, including preoperative diagnostic findings, compartmental excision, histologic diagnosis, and outcome. Three dogs fit the inclusion criteria, which had a sarcoma confined to a single muscular belly (semitendinosus, biceps, and splenius capitis muscles). Complete excision of the affected muscle was performed in all cases. One dog showed moderate lameness in the immediate postoperative period, resulting from the dorsal lifting of the scapula due to serratus ventralis tenotomy performed to remove the caudal insertion of the splenius capitis muscle. All the dogs recovered fully within one month, experiencing good clinical function. Histopathology showed complete tumour removal with no neoplastic fascial disruption in all cases. Compartmental excision provides effective local tumour control, representing an alternative to limb amputation or more radical excision if adjuvant radiotherapy is not an option for owners.

## 1. Introduction

No evidence of distant metastases and definition of the anatomical extent and relationship with surrounding tissues of a tumour is the first step addressed to correct surgical planning finalized to local tumour control.

In human medicine, sarcomas have been classified as intracompartmental or extracompartmental based on their relationship with the fascia (the connective tissue layer tightly connected to the muscle); singular or groups of skeletal muscles covered by a deep fascia can be assimilated into a compartment [1].

More specifically, a compartment is an anatomo-functional unit with the same ontogenic origin and well-defined connective tissue boundaries (i.e., fasciae) [2]. Compartmental excision is based on the principle that a solid tumour that develops inside a compartment tends to grow inside, remaining confined within the fasciae, which act as a barrier [1,3]. Removing only the compartment ensures the same local tumour control as more radical excisions [1,3].The revolutionary concept of compartments was first applied to soft tissue sarcomas in a period in which radiotherapy was not yet widely available [1].

More recently, the same concept has also been applied to both the tumours of the tongue, in which tissue preservation is mandatory to conserve speech and swallowing functions [4,5] and of the lower female genital tract [2].

Compartmental surgery has also been applied in veterinary medicine for solid tumours, considering that surgery may often be the only treatment option accepted by pet owners [6].

### 1.1. Compartmental Surgery in Human Medicine

In the early 1980s, Enneking’s anatomical studies of the compartments of the thigh completely modified the surgical approach to soft tissue sarcomas, underscoring that low-grade, intracompartmental sarcomas could be conservatively removed, thus minimising morbidity and simultaneously obtaining effective local tumour control [1,3].

Enneking divided the body into distinct anatomical sections called compartments and proposed a classification for planning the resection of musculoskeletal tumours based on tumour extension [1,3]. It was hypothesised that some tissues, such as fascia, cortical bone or periosteum, may act as effective barriers against tumour spread. It was observed that low-grade, slow-growing sarcomas tended to follow the path of least resistance without overstepping the fascial boundaries of the compartment [1,3]. If this is true, the complete excision of the compartment results in the complete removal of the tumour, avoiding more crippling and psychologically impacting surgeries such as amputation, as in the case of sarcoma of the limbs.

The local control obtained may not be enough for high-grade soft tissue and/or infiltrative sarcomas in which the fascial layer may be penetrated through perforating vascular channels [3].

Kawaguchi translated anatomical barriers into centimetres of the surgical margin. He divided tissues into thin and thick barriers [7]. A thin barrier is a weaker membranous tissue of muscle fascia, the periosteum of an adult, a vessel sheath, and the epineurium, and it is interpreted as a margin of 2 cm of normal tissue. A thick barrier is represented by a physically strong membranous tissue with a white tendinous lustre, such as a joint capsule and joint cartilage; its complete removal is interpreted as a margin between 3 and 5 cm [7].

Local control is more difficult for lesions in the extracompartmental fascial planes or areas with less distinct fascial boundaries, such as the groin and the popliteal fossa [3].

Additional human studies have validated Enneking’s concepts, even showing an increase in survival times and a lower local recurrence rate compared to patients treated with a more traditional wide-margin excision [8].

Compartmental surgery is not always feasible; in a publication dealing with 143 soft tissue sarcomas [9], only one-third of the tumours were eligible for this approach while, in the remaining cases, they either originated from the subcutis or had already invaded the adjacent compartments. In fact, the site of origin of the tumour within the compartment should be considered. It is rare that the tumour is in the centre of the muscle; more frequently, it expands towards the tendinous origin, or end, of the muscle. In this case, the surgery would be insufficient on the side of the tumour and excessive on the opposite side [9].

In more recent decades, Enneking’s model has been criticised in favour of less invasive approaches, supported by the inclusion of radiotherapy in a multimodal treatment approach, which allows for reducing the surgical dose and, at the same time, lowering the risk of local recurrence [10].

### 1.2. Compartmental Surgery in Veterinary Medicine

The concept of compartmental surgery in humans has often been overcome thanks to the advent of radiotherapy; this may not be true in veterinary medicine due to the high cost of radiotherapy, thus rendering an aggressive malignant tumour excision the unique therapeutic option possible.

Few veterinary studies regarding compartmental tumour excision have been published. The increasing use of computed tomography (CT), which allows a better characterisation of the tumour and its relationship with the surrounding structures, opens up new perspectives for this procedure, thus reducing both the surgical dose and the postoperative morbidity.

At present, the trend in veterinary surgical oncological surgery is to abandon the concept of an “en bloc” resection, always in favour of a more reasoned and planned excision, taking into account the presence and extent of tissue barriers between the tumour and the surrounding tissues.

In 2014, Bray described an anatomically-based surgical technique for a hemipelvectomy that enabled the compartmental resection of tumours in the pelvic region [11]. Better local tumour control was achieved compared with the nearly one-third of the incomplete margins obtained in a Veterinary Society of Surgical Oncology retrospective study in which the “wide margin excisional approach” was applied [12]. Furthermore, anatomical dissection, which involves the removal of the muscle from its origin instead of a mid-belly cut, is usually associated with fewer complications, such as haemorrhage and postoperative pain.

Despite all the limitations and possible confounding factors, Bray’s paper paved the way for the applicability of compartmental surgery, including in veterinary medicine [12].

Nevertheless, compartmental surgery is less frequently applicable in veterinary medicine than in human medicine due to the fact that many mesenchymal neoplasms arise from the subcutis and not from the muscle itself [6]. However, this is not always a limitation, as, in selected cases, an anatomical resection can also be performed in the case of subcutaneous masses by taking advantage of the anatomic barriers represented underneath the muscular compartment [13]. With this in mind, Bray and Polton reported the anatomical resection of one or more compartments in contact with the tumour on 21 cats bearing a feline injection site sarcoma [13]. The compartmental excision avoided a traditional wide-margin excision with less morbidity and an oncological outcome comparable to the more traditional en bloc resection [14,15,16]. Moreover, it has been suggested that the en bloc resection does not consider the mode of spread of skip metastases along the path of least resistance; this may result in an excessive surgical dose at one point and insufficient at another point [13]. In the Bray and Polton paper [13], neoadjuvant epirubicin was used in association with compartmental excision. It should be outlined that the role of neoadjuvant chemotherapy in feline injection-site sarcomas in an attempt to reduce the surgical dose and the resulting tumoral outcome has not been defined yet [17].

An anatomic classification of the relationship between the fascia and the superficial muscles of the neck, trunk [18], and forelimb [19] has been recently reported in dogs. The intent was to provide some guidelines for veterinary surgeons when searching for an adequate deep surgical plane when removing a cutaneous or subcutaneous tumour. In both papers, four types of fasciae were reported: type I, discrete sheets; type II, tightly adherent to thin muscles; type III, tightly adherent to thick muscles; and type IV, associated with the periosteum [18,19]. Additional anatomical studies on the deep muscle fasciae are also required as different types of fasciae may act differently when acting as an anatomic barrier against tumour growth, as has already been reported by Kawaguchi [7]. This new information will be useful in evaluating the outcome of specific tumour histotypes, even of different histological grades.

Compartmental surgery has also been applied to tumours of the peripheral nerves, following the human medicine standard of care, where surgical excision consists of the removal of the affected nerve with a proximal and distal margin; limb amputation is performed only in rare cases [20]. This technique was applied in 16 dogs with brachial plexus peripheral neural sheath tumours (PNST) treated with limb-sparing compartmental resection; 14 of them had an improvement in limb function; in the majority of cases, the excision margins were histologically clean [21].

### 1.3. Aim of the Study

In this case series, the authors reported on the feasibility, technique and outcome of muscular compartmental resection in three dogs bearing an intramuscular soft tissue sarcoma.

## 2. Materials and Methods

### 2.1. Case Selection

The medical records of dogs referred to the Veterinary Teaching Hospital (VTH) of the Department of Veterinary Sciences of the University of Turin (Turin, Italy) for intramuscular sarcomas from January 2018 to June 2022 were identified and reviewed.

The inclusion criteria were: (a) the presence of a muscular sarcoma, (b) complete tumour staging to exclude distant tumour spread and confirm the integrity of the muscular fasciae (intramuscular sarcoma); (c) complete muscular compartmental excision; (d) complete histological report; (e) a minimum of a 30-days follow-up.

Written consent was obtained from the owners for all the procedures [preoperative cytology, preoperative work-up including blood and urine exams, cardiologic examination, preoperative incisional biopsy (if performed), anaesthesia, total body CT, surgical excision, and postoperative histology] before proceeding. Perioperative standard-of-care management, including analgesia, was assured for all the dogs. This study did not fall within the application areas of Italian Legislative Decree 26/2014, which governs the protection of animals used for scientific or educational purposes. Therefore, ethical approval was waived for this study. The animals were not treated as part of an experimental study; only the data were later selected and included in this study. No specific informed consent statement was signed for inclusion in this retrospective study.

### 2.2. Surgery

The dogs were premedicated with an intra-muscular combination of dexmedetomidine (2 mcg/kg) (Dexdomitor^®^, Orion Pharma, Turku, Finland) and methadone (0.2 mg/kg) (Synthadon^®^, Le Vet Beheer B.V., Oudewater, The Netherlands). General anaesthesia was induced with propofol (3 mg/kg, IV) (Proposure^®^, Merial Italia Spa, Noventa Padovana, Italy) and maintained with isoflurane in oxygen (Isoflo; Esteve Spa, Barcelona, Spain). After being clipped and aseptically prepared, the patients were moved to the surgical room and positioned with the affected muscles entirely included within the surgical field. Muscular excision was performed in order to remove the complete muscular belly with both tendons. The intravenous administration of cefazolin (20 mg/kg) (Cefazolina Teva, Teva Italia SRL, Milano, Italy) was performed 20 min before skin incision and repeated every 60 min until extubation.

### 2.3. Histopathology

Histological diagnosis on haematoxylin and eosin staining was performed by pathologists at the Department of Veterinary Science, University of Turin, according to Roccabianca et al. [22]. Microscopic evaluation of margins consisted of a qualitative margin assessment on fascial planes and tumour borders as previously described [7]. The absence of tumour cells at the surgical margin was categorised as “non-infiltrated” margins; tumour cells at or within the proximity of several cell layers at the surgical margin without the presence of a fascial plane were defined as “infiltrated margins”.

### 2.4. Post-Operative Care and Follow-Up

The postoperative management included intravenous fluid (lactated Ringer’s 2 mL/kg/h), analgesia with methadone (Semfortan^®^, Eurovet Animal Health B.V., Bladel, The Netherlands; 0.5 mg/kg IV q8h) or buprenorphine (Temgesic^®^, Schering-Plough, Segrate, Italy; 0.3 mcg/kg IV q12 h), and meloxicam (Metacam^®^, Boehringer Ingelheim, Ingelheim/Rhein, Germany; 0.2 mg/kg IV the first day and 0.1 mg/kg IV thereafter). The dogs were all discharged with an Elizabethan collar and meloxicam (Metacam^®^, Boehringer Ingelheim, Ingelheim/Rhein, Germany: 0.1 mg/kg daily for seven days, PO) within 1–5 days and were then re-evaluated on days 7, 14 and 21 after discharge.

Complications have been classified accordingly to the guidelines of “Veterinary Cooperative Oncology Group—Common Terminology Criteria for Adverse Events” (VCOG-CTCAE) [23]. Follow-up data on limb use, disease-free interval and survival was acquired by periodic examination or by direct communication with owners and referring veterinarians. Based on the final histopathological diagnosis, the need for chemotherapy was evaluated for every case.

## 3. Results

Three dogs bearing intramuscular sarcoma met the inclusion criteria and are described in this study.

### 3.1. Case 1

**Figure 1:**
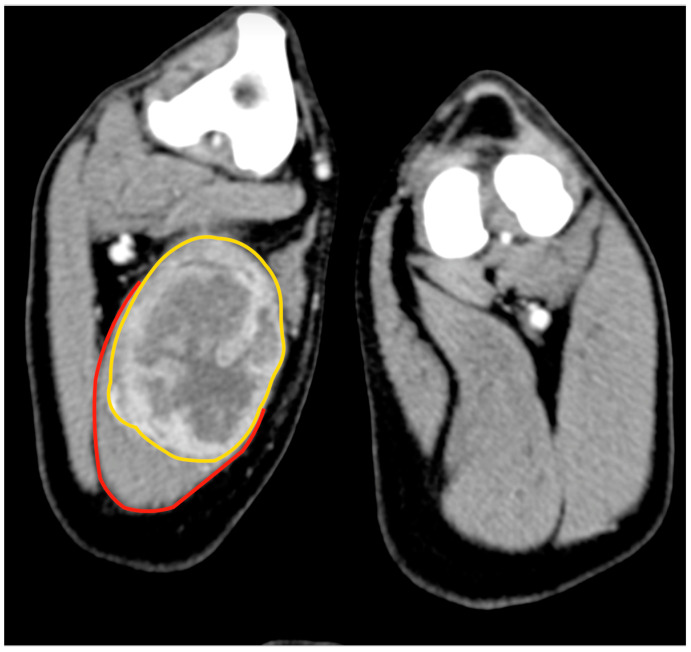
Tomographic appearance of the neoplasm within the semitendinous muscle. The yellow line delimitates the mass, and the red line is the semitendineous muscle. Orientation: cranial is on the top of the figure.

An 11.5-year-old, 28 kg, castrated male Labrador retriever was referred for a 4 cm painless, firm mass in the caudal region of the thigh. The mass had been present for several months but had recently grown. The dog showed no lameness or discomfort. Physical examination and the laboratory profile (complete blood count—CBC, biochemical values and urinalysis) were unremarkable. An incisional punch biopsy was performed during the total body CT exam (Figure 1). The histology was compatible with a soft tissue sarcoma showing chondromyxoid differentiation.

For the surgical excision, the dog was positioned in the left lateral recumbency. A skin incision was made from the ischiatic tuberosity to the popliteal region, with an elliptical deviation of 3 cm around the biopsy site. The subcutis underwent blunt dissection, and Farabeuf retractors were used to retract the skin edges laterally and medially in order to visualise the underlying muscle bellies. The semitendinosus muscle, with its medio-distally located mass, was isolated from the biceps muscle laterally and the semimembranosus muscle medially. Its attachment on the ischiatic tuberosity was resected using a number 11 surgical blade. The proximal and distal vascular pedicles (a branch of the caudal gluteal artery proximally and a branch of the distal caudal femoral artery distally) were electrocoagulated. Passing the popliteal fossa, a periosteal elevator was used to transect the semitendinosus attachment on the medial surface of the tibia. Walking sutures were placed before wound closure using 2-0 absorbable monofilament to limit the dead space before skin closure. A modified Robert Jones bandage was applied for 48 h.

The dog was able to walk with the aid of a rear suspensor 12 h after surgery. No seroma formation or other complications were recorded during the hospitalisation period, and the dog was discharged 24 h postoperatively. At the 1-week postoperative check-up, the dog presented VCOG-CTCAE grade 1 lameness [23]. Neither seroma formation nor other complications were observed, except for a slight redness of the wound, due to licking. The lameness resolved approximately 12 days after surgery. The dog regained complete function of the limb within one month, being able to run and jump. Definitive histological evaluation was compatible with a grade II chondrosarcoma; the excisional margins were free of neoplastic cells.

Five months postoperatively, the dog was examined for VCOG-CTCAE grade 3 lameness [23] affecting the right front limb; radiological examinations revealed a lytic lesion on the distal scapula and a pulmonary mass. The cytology of the scapular lesion was consistent with a malignant epithelial tumour; the owners refused additional investigation and opted for euthanasia.

### 3.2. Case 2

An 11-year-old, 19 kg, spayed female Bloodhound was admitted for a perivascular wall tumour (PWT) recurrence localised at the level of the caudal aspect of the thigh. The tumour had been removed three years earlier by the referring veterinarian. The recurrence appeared as a thickening of the scar of the first surgery, which deepened into the underlying muscular tissue. The remaining clinical examination was unremarkable, with the exception of a slightly decreased body condition score (2/5), and a CBC showing mild neutrophilia.

**Figure 2:**
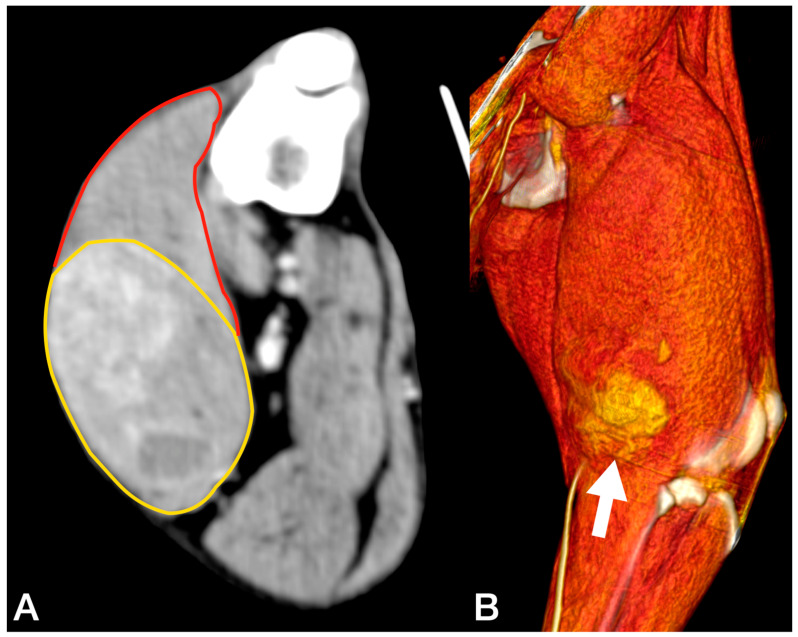
Tomographic appearance of the sarcoma within the biceps femoris muscle, axial view (**A**). The yellow line delimitates the tumours, the red line the biceps muscle 3D reconstruction, and the white arrow indicates the tumour (**B**).

Cytology of the lesion via fine needle aspiration (FNA) was not diagnostic; therefore, an incisional biopsy was performed at the time of the total body CT. This revealed a 5.8 cm × 5.5 cm, poorly defined mass confined within the biceps femoris muscle, with heterogeneous enhancement after the administration of an intravenous contrast medium (Figure 2). No lesions suggestive of metastatic spread were observed. The histology from the bioptic sample was morphologically suggestive of PWT.

**Figure 3:**
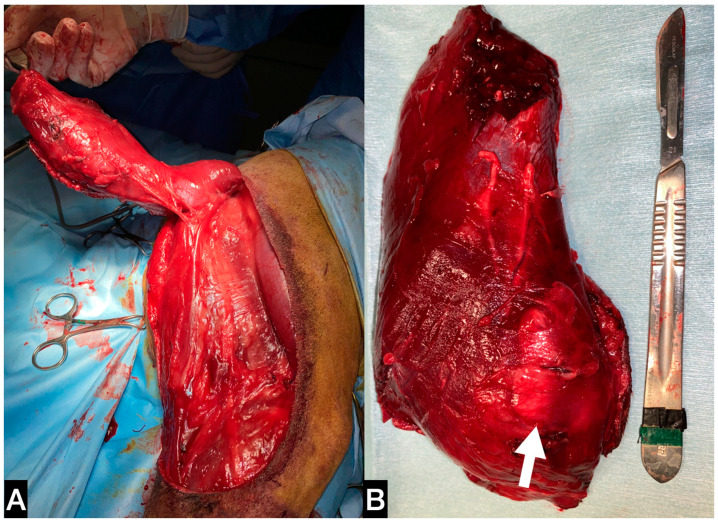
(**A**) Intra-operative picture of the detachment of the biceps from distal to proximal. (**B**) Medial aspect of the anatomic sample, the tumour is confined within the fascial layer, marked with white arrow.

The dog was positioned in left lateral recumbency, and an elliptical skin incision was performed with 3 cm margins around the biopsy site on the lateral surface of the thigh. The incision was then extended proximally to the ischiatic tuberosity and distally to the tibial crest. The subcutaneous fat and the superficial fascia were incised under the skin incision. After dissecting the fascia lata, the biceps muscle was isolated from the vastus lateralis muscle. The craniodistal insertion of the biceps was isolated and resected from the craniolateral aspect of the tibial crest. Finally, the biceps muscle was freed from the mid-proximal femur, dissecting the fibres adherent to the bone (Figure 3A).

A tenotomy was performed at the level of the lateral border of the ischiatic tuberosity to definitively remove the muscle. The deep wound was sutured in a continuous pattern, as previously described. A modified Robert Jones bandage was applied for 48 h

**Figure 4:**
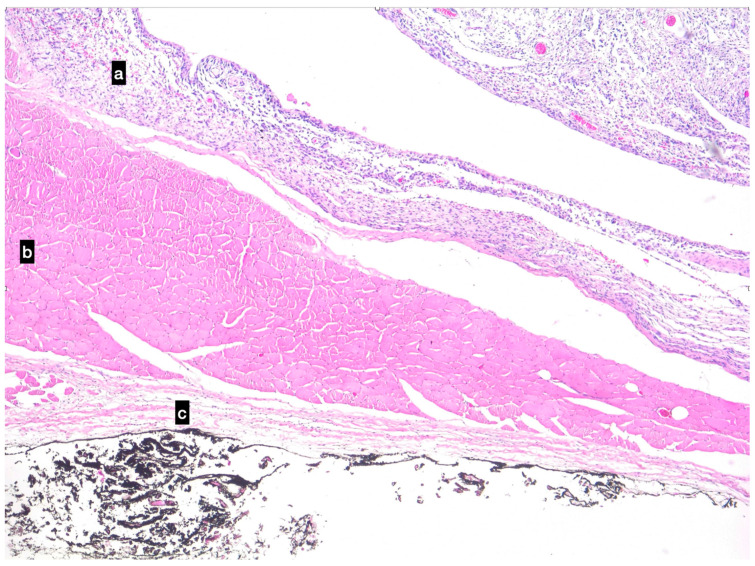
Photomicrographs of the perivascular wall tumour in the upper part of the image (**a**), confined to the muscular belly of the biceps (**b**) in the bottom part of the image, the fascial layer is intact (**c**). The black ink represents the deep surgical margin. Imaging was conducted using a Haematoxylin and eosin stain.

The dog was able to walk 24 h after the procedure. The modified Robert-Jones bandage was removed 48 h after surgery at the time of discharge. The patient was evaluated seven days postoperatively. At this check-up, the dog showed VCOG-CTCAE grade 1 lameness [23] and a mild serous discharge from the wound; however, no medication was required, and it resolved spontaneously within five days. At the 21-day postoperative check-up, the lameness was no longer apparent. A cosmetic defect remained due to the lack of the biceps muscle in the postero-lateral aspect of the thigh. Definitive histological evaluation was compatible with a grade I PWT (Figure 4). The dog is alive 900 days after surgery, and no signs of recurrence have been detected.

### 3.3. Case 3

**Figure 5:**
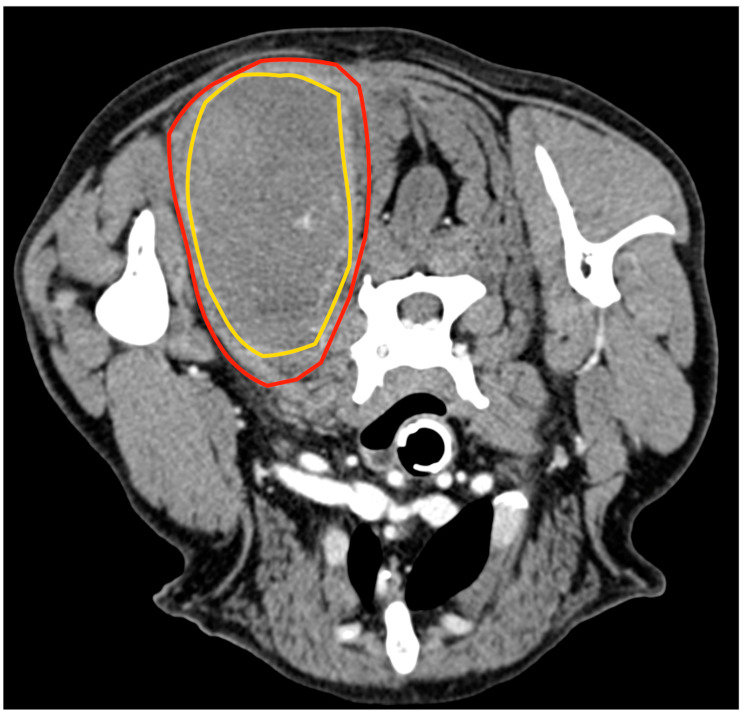
Tomographic appearance of the neoplasm within the splenius capitis muscle between the seventh cervical vertebra and the medial surface of the scapula. The yellow line delimitates the tumours, and the red line is the splenius capitis muscle.

An 8-year-old 35 kg spayed female mixed breed was referred for a 10 cm mass localised between the dorsal neck and the left scapular region, which had been noted a week earlier and had grown rapidly. At clinical examination, the mass appeared as a painful, firm, subcutaneous lesion adhering to the underlying tissues. No regional lymphadenopathy was detected. The cytology performed by the referring veterinarian was indicative of a malignant mesenchymal sarcoma. Total body CT confirmed the anatomic confinement of the tumour to the dorsal surface of the splenius capitis (Figure 5). No other anomalies were found based on the CT exam.

**Figure 6:**
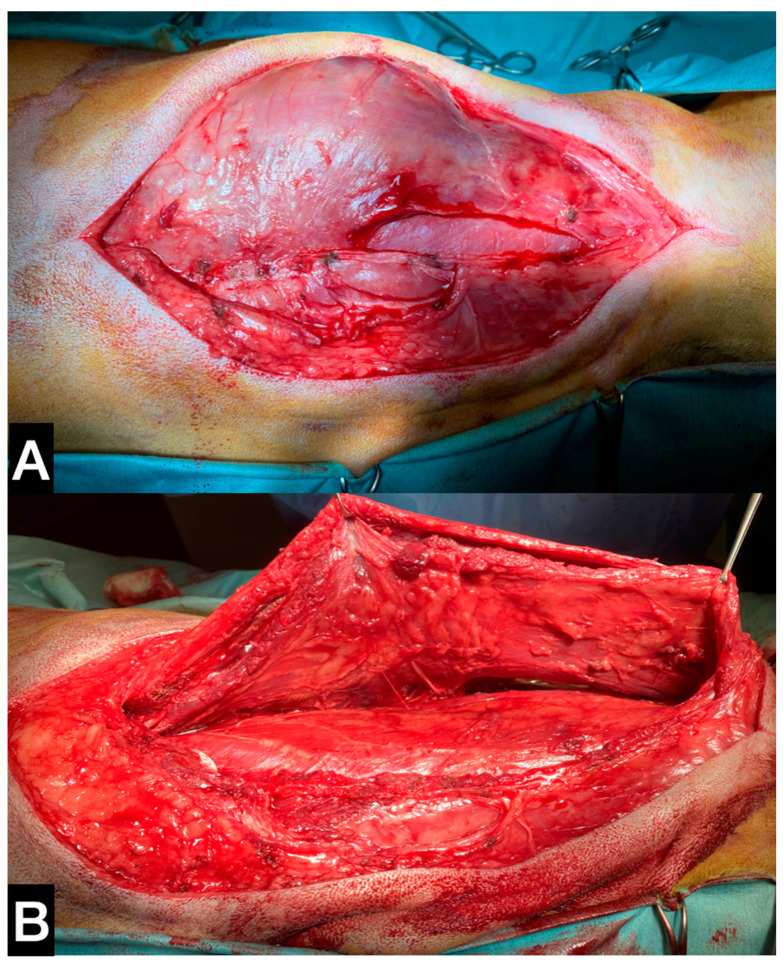
(**A**) Surgical paramedian approach to the splenius. (**B**) The muscle is released from the spinous process on the median side. Figure orientation the head of the dog is on the right side of the picture.

With the patient in sternal recumbency but with a mild rotation to the right side, a skin incision from the external occipital protuberance to the spinous process of the fifth thoracic vertebra was performed (Figure 6A).

The splenius capitis was isolated dorsally from the rhomboideus and distally from the serratus ventralis muscles. Caudally, a partial tenotomy of the aponeurosis of the serratus dorsalis cranialis muscle was performed in order to visualise and remove the caudal insertion of the splenius from the third thoracic vertebra. Cranially, the splenius muscle was dissected from the occipital protuberance. The muscle was completely isolated and excised by dissecting its belly from the dorsolateral aspect of the cervical and thoracic vertebrae (Figure 6B). The partial tenotomy of the serratus dorsalis was sutured using a continuous suture pattern. The fascial planes were sutured using absorbable monofilament, and an active drain was placed as the splenius removal resulted in a large dead space. A soft thoracic bandage was applied for seven days.

The patient was able to stand and walk 12 h after surgery, but a VCOG-CTCAE grade 2 lameness was present due to the dorsal lifting of the scapula during movement. The active drain was removed five days after surgery when the dog was discharged with a soft thoracic bandage.

At the 7-day check-up, the thoracic bandage was removed. The wound appeared normal, and no seroma was observed. Fourteen days after surgery, the patient appeared painless during walking; however, the mechanical lameness on the left thoracic limb persisted (VCOG-CTCAE grade 1 lameness). The left scapula tended to rise dorsally with each step due to the lack of attachment of the splenius on the cervical and thoracic vertebrae. This anomalous motion decreased over time and was almost imperceptible after two months.

The histological report was consistent with a subcutaneous intramuscular hemangiosarcoma. The excisional margins were free of neoplastic cells. Chemotherapy was started 20 days after surgery (doxorubicin [30 mg/m^2^]) given by slow intravenous infusion every three weeks; after four cycles of doxorubicin, metronomic therapy was instituted (thalidomide 4 mg/kg and chlorambucile 4 mg/m^2^ PO SID). The dog was euthanized 183 days after surgery for seizures unresponsive to therapy due to brain metastases.

## 4. Discussion

With this limited case series, the authors attempted to show the feasibility of effective local control of selected intramuscular neoplasms by completely removing the muscle and its fascia. For cases of muscular sarcomas of the limbs, compartmental surgery should be considered as both a less invasive and a limb-salvage procedure as compared to limb amputation. Compartmental excision is feasible if the integrity of the fascia is maintained, as suggested by Kawaguchi [7], thus resulting in complete local tumour control due to the presence of anatomical barriers and, at the same time, with the advantage of removing less healthy tissue. Limb amputation (or other aggressive procedures for tumours of other localisations) should be reserved for soft tissue sarcomas that cannot be completely excised with adequate margins or are intercompartmental, i.e., those in which the removal would cause a substantial-to-complete loss of limb function.

The recent anatomic classification of the superficial muscular fasciae [18,19] cannot be applied to the present study because both studies do not take into account intermuscular and deep fasciae. For example, the biceps femoris muscle is characterised by multiple types of fasciae: superficially, there is a type III fascia (tightly adherent to thick muscles), while caudo-medially, there is the bone insertion, thus being a type IV (i.e., associated with the periosteum). Depending on the intramuscular location of the tumour, the type of fascial border could be different along the muscular belly. Further anatomical studies are needed to improve deep fascial knowledge to be applied to tumour resection in veterinary medicine.

Functional recovery was quick and complete in all cases; this could be explained by the limited surgical invasiveness resulting from the excision of a single muscle that can be substituted by other agonist muscles of the same region. A compartmental excision should be avoided in the case of the involvement of muscles, the function of which cannot be substituted, such as the rectus femoris or the triceps brachii. In this scenario, the biceps femoris muscle has three functions; it acts as a hip extensor, stifle flexor and tarsal extensor as part of the common calcaneal action [24]. These actions are also accomplished by the semitendinosus and semimembranosus muscles, and the remaining calcanean tendon, with a rapid return to weight-bearing and functional limb usage after biceps muscle excision [25]. Indeed, the excision of the biceps femoris has already been described, and authors reported a good oncologic and functional outcome, with only one dog experiencing wound dehiscence caused by tension related to the large amount of skin removed [25]. Regarding the excision of the semitendinosus muscle, it is already known that it can be completely transposed (for perineal hernia) without causing any functional deficit [26,27]. The main actions of the semitendinosus muscle are the extension of both hip and tarsal joints; these actions can be partially replaced by the semimembranosus (hip extension) and the other muscles forming the common calcanean tendon (tarsal extension) [24]. Regarding the splenius capitis, its action is to extend, raise and draw laterally the head and neck [24]. These functions can be substituted by the longissimus cervicis muscle.

As physical examination alone cannot estimate the real anatomic localization and extension of deeper tumours, the use of a second level of diagnosis (CT scan and/or Magnetic Resonance) is mandatory [28].

Magnetic Resonance (MR) imaging is a more reliable tool than a CT scan for the characterization of soft tissue sarcoma, even though CT displays some limitations on the soft tissue evaluation. In human medicine, MR features are relevant for the assessment of treatment strategies, surgical planning and definition of borders/signs of fascial infiltration [29]. Despite this, CT remains a common secondary-level diagnostic tool often used in veterinary medicine due to either higher costs or less equipment availability. Advanced imaging, as part of tumour staging, allows for defining which specific muscle the tumour is located in and ascertaining the macroscopic integrity of the muscular fascia. Additional information regarding the integrity of the fascia can be provided by ultrasonography. The fascia generally appears as a linear hyperechoic structure with boundaries easily identifiable due to the adjacent hypoechoic muscles [30]. However, ultrasound has two major limitations: only superficial muscles can be evaluated, and it provides a subjective evaluation.

Local control of low to intermediate-grade malignant tumours can be achieved with surgery alone or with surgery and adjuvant radiotherapy. When histopathology is consistent with a high-grade tumour, adjuvant chemotherapy is needed despite negative tumour clinical staging at presentation and completeness of excision margins. Muscular hemangiosarcoma is a high-grade sarcoma due to its high metastatic rate, and adjuvant chemotherapy is considered essential in an attempt to prolong survival time [31]. In this case no other options, but surgery, were available for obtaining local tumour control.

Even if the compartmental excision can be classified, when applied to tumours of the limbs, as less invasive than amputation, nevertheless it should be considered a major elective procedure, which requires good anatomical knowledge of the region of interest; the goal is to avoid any vascular or neurological damage. Additionally, the complete excision of a muscle may result in large dead spaces and, consequently, in seroma formation. In this limited cases series, only in dog number 3 an active draining system was applied, while in the remaining two dogs, only a soft padded bandage was applied for 48 h, but no seroma formation occurred even though a draining system was not applied. Despite this, it is reasonable to state that it would be prudential to apply it in all cases.

Postoperative complications observed were few. All dogs experienced a transient and self-limiting lameness. In two dogs, the lameness was caused by the pain secondary to soft tissue surgical trauma. On the contrary, the cause of the persistent lameness in case number 3 was mechanical due to the tenotomy of the serratus ventralis tenotomy. Dorsal scapular luxation is a common consequence of the traumatic rupture of the serratus ventralis muscle [32]. This condition can be treated surgically or conservatively with bandage application [33]. As the tenotomy was partial in this case, the authors decided to apply a conservative treatment; the dog regained normal function in two months. Dehiscence did not occur in any of the dogs in the present series as the intramuscular localisation of the tumour always allowed sparing the skin with the exception of that corresponding to the biopsy site that was removed with a 3-cm margin around (Cases 1 and 2). Care should be taken, especially when the compartmental excision of a tumour is an available option, to choose a proper biopsy site since it needs to be removed with the compartment underneath [34].

The main limitations of this report are the low number and the heterogeneity of the cases included and its retrospective nature. The first condition is influenced by the rarity of the disease in dogs, i.e., patients with soft tissue sarcoma limited to a single muscle belly. Nevertheless, this report provides some guidelines regarding the selection of patients who could potentially be candidates for a compartmental excision. When this selection is correct, it is possible to reach a local tumour control by performing a less aggressive surgery, often resulting in a desirable preservation of function.

Overall, it can be concluded that dogs with mid-belly intramuscular sarcomas still confined within the muscular fascia are potential candidates for a compartmental excision. Attention should be addressed to the resulting function; this requires in-depth knowledge of both anatomy and physiology of the region.

## 5. Conclusions

The main advantages of a compartmental excision are the possibility of obtaining local tumour control and function preservation. While in humans, these goals may also be reached with marginal excision plus radiotherapy or radiotherapy only, in veterinary medicine, radiotherapy, despite being a well-known therapeutic option, is not always used due to its high cost and/or equipment availability. With this in mind, in case of an intramuscular soft tissue sarcoma still confined to a single and not functionally essential muscle, a compartmental excision may be a valuable option.

## Data Availability

Authors declare that data associated within this paper are accessible and available.

## References

[1] Enneking W.F., Spanier S.S., Goodman M.A. (1980). A system for the surgical staging of musculoskeletal sarcoma. Clin. Orthop. Relat. Res..

[2] Hockel M. (2012). Cancer permeates locally within ontogenetic compartments: Clinical evidence and implications for cancer surgery. Future Oncol..

[3] Enneking W.F., Spanier S.S., Malawer M.M. (1981). The effect of the anatomic setting on the results of surgical procedures for soft parts sarcoma of the thigh. Cancer.

[4] Calabrese L., Giugliano G., Bruschini R., Ansarin M., Navach V., Grosso E., Gibelli B., Ostuni A., Chiesa F. (2009). Compartmental surgery in tongue tumours: Description of a new surgical technique. Acta Otorhinolaryngol. Ital..

[5] Ansarin M., Bruschini R., Navach V., Giugliano G., Calabrese L., Chiesa F., Medina J.E., Kowalski L.P., Shah J.P. (2019). Classification of GLOSSECTOMIES: Proposal for tongue cancer resections. Head Neck.

[6] Bray J.P. (2017). Soft tissue sarcoma in the dog—Part 2: Surgical margins, controversies, and a comparative review. J. Small Anim. Pract..

[7] Kawaguchi N., Ahmed A.R., Matsumoto S., Manabe J., Matsushita Y. (2004). The concept of curative margin in surgery for bone and soft tissue sarcoma. Clin. Orthop. Relat. Res..

[8] Azzarelli A. (1993). Surgery in soft tissue sarcomas. Eur. J. Cancer.

[9] Stotter A., Fallowfield M., Mott A., Fisher C., Westbury G. (1990). Role of compartmental resection for soft tissue sarcoma of the limb and limb girdle. J. Br. Surg..

[10] Miller E., Xu-Welliver D., Haglund K.E. (2015). The role of modern radiation therapy in the management of extremity sarcomas. J. Surg. Oncol..

[11] Bray J.P. (2014). Hemipelvectomy: Modified Surgical Technique and Clinical Experiences from a Retrospective Study. Vet. Surg..

[12] Bray J.P., Worley D.R., Henderson R.A., Boston S.E., Mathews K.G., Romanelli G., Bacon N.J., Liptak J.M., Scase T.J. (2013). Hemipelvectomy: Outcome in 84 dogs and 16 cats. A Veterinary Society of Surgical Oncology Retrospective study. Vet. Surg..

[13] Bray J.P., Polton G. (2014). Neoadjuvant and adjuvant chemotherapy combined with anatomical resection of feline injection-site sarcoma: Results in 21 cats. Vet. Comp. Oncol..

[14] Martano M., Morello E., Buracco P. (2011). Feline injection-site sarcoma: Past, present and future perspectives. Vet. J..

[15] Phelps H.A., Kuntz C.A., Milner R.J., Powers B.E., Bacon N.J. (2011). Radical excision with five-centimeter margins for treatment of feline injection-site sarcomas: 91 cases (1998–2002). J. Am. Vet. Med. Assoc..

[16] Zabielska-Koczywas K., Wojtalewicz A., Lechowski R. (2017). Current knowledge on feline injection-site sarcoma treatment. Acta Vet. Scand..

[17] Martano M., Morello E., Ughetto M., Iussich S., Petterino C., Cascio P., Buracco P. (2005). Surgery alone versus surgery and doxorubicin for the treatment of feline injection site sarcomas: A report of 69 cases. Vet. J..

[18] Schroeder M.M., Skinner O.T. (2022). Fascial plane mapping for superficial tumor resection in dogs. Part I: Neck and Trunk. Vet. Surg..

[19] Latifi M., Skinner O.T., Schroeder M.M., Mickelson M.A. (2022). Fascial plane mapping for superficial tumor resection in dogs. Part II: Forelimb. Vet. Surg..

[20] Zou C., Smith K.D., Liu J., Lahat G., Myers S., Wang W.L., Zhang W., McCutcheon I.E., Slopis J.M., Lazzar A.J. (2009). Clinical, pathological, and molecular variables predictive of malignant peripheral nerve sheath tumor outcome. Ann. Surg..

[21] Van Stee L., Boston S., Teske E., Meij B. (2017). Compartmental resection of peripheral nerve tumours with limb preservation in 16 dogs (1995–2011). Vet. J..

[22] Roccabianca P., Schulman F.Y., Avallone G., Foster R.A., Scruggs J.L., Dittmer K., Kiupe M. (2020). Surgical Pathology of Tumors of Domestic Animals 3: Tumors of Soft Tissue.

[23] LeBlanc A.K., Atherton M., Bentley R.T., Boudreau C.E., Burton J.H., Curran K.M., Dow S., Giuffrida M.A., Kellihan H.B., Mason N.J. (2021). Veterinary Cooperative Oncology Group-Common Terminology Criteria for Adverse Events (VCOG-CTCAE v2) following investigational therapy in dogs and cats. Vet. Comp. Oncol..

[24] Evans H.E., de Lahunta A. (2012). Miller’s Anatomy of the Dog.

[25] Connery N.A., Bellenger C.R. (2002). Surgical management of haemangiopericytoma involving the biceps femoris muscle in four dogs. J. Small Anim. Pract..

[26] Chambers J.N., Rawlings C.A. (1991). Applications of a semitendinosus muscle flap in two dogs. J. Am. Vet. Med. Assoc..

[27] Mortari A.C., Rahal S.C., Resende L.A., Dal-Pai-Silva M., Mamprim M.J., Correa M.A., Antunes S.H. (2005). Electromyographical, ultrasonographical and morphological modifications in semitendinous muscle after transposition as ventral perineal muscle flap. J. Vet. Med. Ser. A.

[28] Ferrari R., Di Giancamillo M., Stefanello D., Giudice C., Grieco V., Longo M., Ravasio G., Boracchi P. (2017). Clinical and computed tomography tumours dimension assessments for planning wide excision of injection site sarcomas in cats: How strong is the agreement?. Vet. Comp. Oncol..

[29] Scalas G., Parmeggiani A., Martella C., Tuzzato G., Bianchi G., Facchini G., Clinca R., Spinnato P. (2021). Magnetic resonance imaging of soft tissue sarcoma: Features related to prognosis. Eur. J. Orthop. Surg. Traumatol..

[30] Fede C., Gaudreault N., Fan C., Macchi V., De Caro R., Stecco C. (2018). Morphometric and dynamic measurements of muscular fascia in healthy individuals using ultrasound imaging: A summary of the discrepancies and gaps in the current literature. Surg. Radiol. Anat..

[31] Bulakowski E.J., Philibert J.C., Siegel S., Clifford C.A., Risbon R., Zivin K., Cronin K.L. (2008). Evaluation of outcome associated with subcutaneous and intramuscular hemangiosarcoma treated with adjuvant doxorubicin in dogs: 21 cases (2001–2006). J. Am. Vet. Med. Assoc..

[32] Jones S.C., Tinga S., Porter E.G., Lewis D.D. (2017). Surgical management of dorsal scapular luxation in three dogs. Vet. Comp. Orthop. Traumatol..

[33] Piermattei D.L., Flo G., De Camp C. (2006). The Shoulder Joint. Brinker, Piermattei and Flo’s Handbook of Small Animal Orthopedics and Fracture Repair.

[34] Withrow S. (1991). The three rules of good oncology: Biopsy, biopsy, biopsy. J. Am. Anim. Hosp. Assoc..

